# Efficacy and Safety of Sitagliptin for the Treatment of New-Onset Diabetes after Renal Transplantation

**DOI:** 10.1155/2014/617638

**Published:** 2014-04-10

**Authors:** Brian P. Boerner, Clifford D. Miles, Vijay Shivaswamy

**Affiliations:** ^1^Department of Internal Medicine, University of Nebraska Medical Center, 984130 Nebraska Medical Center, Omaha, NE 68198, USA; ^2^VA Nebraska-Western Iowa Health Care System, University of Nebraska Medical Center, 4101 Woolworth Avenue, Omaha, NE 68105, USA

## Abstract

New-onset diabetes after transplantation (NODAT) is a common comorbidity after renal transplantation. Though metformin is the first-line agent for the treatment of type 2 diabetes, in renal transplant recipients, metformin is frequently avoided due to concerns about renal dysfunction and risk for lactic acidosis. Therefore, alternative first-line agents for the treatment of NODAT in renal transplant recipients are needed. Sitagliptin, a dipeptidyl-peptidase-4 (DPP-4) inhibitor, has a low incidence of hypoglycemia, is weight neutral, and, in a small study, did not affect immunosuppressant levels. However, long-term sitagliptin use for the treatment of NODAT in kidney transplant recipients has not been studied. We retrospectively analyzed renal transplant recipients diagnosed with NODAT and treated with sitagliptin to assess safety and efficacy. Twenty-two patients were started on sitagliptin alone. After 12 months of followup, 19/22 patients remained on sitagliptin alone with a significant improvement in hemoglobin A1c. Renal function and immunosuppressant levels remained stable. Analysis of long-term followup (32.5 ± 17.8 months) revealed that 17/22 patients remained on sitagliptin (mean hemoglobin A1c < 7%) with 9/17 patients remaining on sitagliptin alone. Transplant-specific adverse events were rare. Sitagliptin appears safe and efficacious for the treatment of NODAT in kidney transplant recipients.

## 1. Introduction

New-onset diabetes after transplantation (NODAT) represents a common comorbidity after solid organ transplantation. NODAT after kidney transplantation is associated with inferior outcomes, including higher risk of infections, decreased graft survival, higher rates of cardiovascular disease, and increased overall mortality [1–5]. Risk factors for NODAT include use of immunosuppressants, in particular corticosteroids and calcineurin-inhibitors, as well as traditional risk factors for type 2 diabetes including obesity, family history, age, and ethnicity [6–8].

Metformin is the first-line agent of choice for the treatment of type 2 diabetes in the general population. Though some data suggests metformin is safe in the setting of chronic kidney disease, the use of metformin in kidney transplant patients is still quite controversial due to the concern about lactic acidosis and lack of data regarding metformin use in this patient population [9–11]. Therefore, it is important to identify an alternative first-line oral agent for the treatment of NODAT in kidney transplant recipients. Short-term studies have shown some oral antihyperglycemic agents to be effective in patients with NODAT [12–14]. However, use of these agents is limited by adverse effects including fluid retention and bone loss with pioglitazone and hypoglycemia and weight gain with insulin secretagogues [12, 15, 16].

Sitagliptin, an oral dipeptidyl-peptidase-4 (DPP-4) inhibitor, was approved in the United States in 2006 for the treatment of type 2 diabetes. Sitagliptin has a low incidence of hypoglycemia and does not promote weight gain, and the dosage can be adjusted based on renal function [17]. These properties make this medication an attractive option for the treatment of NODAT. Additionally, in a 3-month trial of kidney transplant recipients, sitagliptin use did not alter immunosuppressant levels [18]. However, evidence supporting the efficacy and safety of long-term use of sitagliptin in kidney transplant recipients is lacking.

We hypothesized that in kidney transplant recipients sitagliptin would improve NODAT control, as assessed by hemoglobin A1c (HbA1c), with a favorable safety profile including no effect on immunosuppressant levels or graft function.

## 2. Materials and Methods

In this single center study, we performed a retrospective analysis of kidney transplant recipients in the Multidisciplinary Transplant Clinic at the University of Nebraska Medical Center (UNMC) between October 2006 (release date of sitagliptin in the United States) and December 2012. All subjects in this study received their kidney transplant at UNMC. Between 2009 and 2013, 125 to 149 kidney transplants were performed per year at UNMC (http://optn.transplant.hrsa.gov/). We have previously reported an incidence of NODAT of 18% in our kidney transplant population [19].

The study was approved and monitored by the UNMC Institutional Review Board. A general query was performed to identify all kidney transplant recipients who had been prescribed sitagliptin during the study dates. Data for the query and subsequent data collection for the study were obtained from review of the electronic medical records—Centricity (GE Healthcare), OTTR (OTTR Chronic Care Solutions), and Epic (Epic Systems Corporation). Inclusion criteria for the study included diagnosis of NODAT by the 2003 International Consensus Guidelines criteria (fasting plasma glucose ≥126 mg/dL or 7.0 mmol/L after no caloric intake for at least 8 hours* or *random plasma glucose ≥200 mg/dL or 11.1 mmol/L with symptoms of diabetes* or *2 h plasma glucose in a 75 g oral glucose tolerance test (OGTT) ≥200 mg/dL or 11.1 mmol/L) and diabetes care received at the UNMC Diabetes Center to allow for accurate record of diabetes medication changes and reporting of side effects [20]. Additionally, to specifically understand the efficacy and safety of sitagliptin for the treatment of NODAT, patients included in the study either used sitagliptin as the initial agent for NODAT or had other diabetes medications stopped prior to or at the time of sitagliptin initiation. This allowed a period of time to analyze patients on sitagliptin alone. Exclusion criteria included diagnosis of diabetes prior to transplant and death or loss of followup prior to 12 months after initiation of sitagliptin. Sitagliptin dosing was performed based on the patients' renal function, as directed by the prescribing information for sitagliptin (https://www.merck.com/).

Initiation of sitagliptin therapy marked the start of followup for each patient and patients were followed up until discontinuation of sitagliptin or December 2012, whichever came first. Two endpoints were established. The first was a 12-month followup to assess the subacute efficacy and safety of sitagliptin in terms of diabetes control, side effects, immunosuppressant levels, and graft function. The second endpoint was at discontinuation of sitagliptin or, for those patients who continued on sitagliptin through the entire study, in December 2012. This longer followup allowed for assessment of long-term efficacy and safety in terms of diabetes control, acute rejection episodes, allograft function, and other side effects. The patient medical records were reviewed in detail for a number of different diabetes and transplant-specific outcomes including dates of sitagliptin initiation/discontinuation; dates of initiation/discontinuation of any other diabetes medications; patient or physician-reported side effects; reason for discontinuation of sitagliptin; acute rejection episodes; graft loss; immunosuppressant regimens and dosages; opportunistic infections; and death. Body mass index (BMI) was obtained at the time of transplant, at the time of sitagliptin initiation, after 12 months of sitagliptin therapy, and at the end of followup.

As part of routine posttransplant and diabetes care, patients underwent fasting labs weekly to monthly at the Clinical Laboratory of The Nebraska Medical Center including serum creatinine, tacrolimus and/or sirolimus trough levels (as appropriate), and fasting glucose levels. HbA1c was performed every 3 months and fasting lipid panel every 3 to 6 months. Serum creatinine, fasting serum glucose, HDL cholesterol, and LDL cholesterol measurements were performed using standard calibration protocols and dedicated analyzers and were assayed by colorimetric means. Tacrolimus and sirolimus levels were performed via liquid chromatography/tandem mass spectrometry. HbA1c was performed on a National Glycohemoglobin Standardization Program (NGSP) certified high-pressure liquid chromatography (HPLC) analyzer. Estimated GFR (eGFR) was calculated using the Modification of Diet in Renal Disease (MDRD) equation.

Data is presented as mean ± SD. Paired groups were analyzed utilizing the Wilcoxon Signed Rank test. Nonparametric repeated measures ANOVA was used to analyze parameters at multiple time points. *P* < 0.05 was considered significant.

## 3. Results

**Figure 1:**
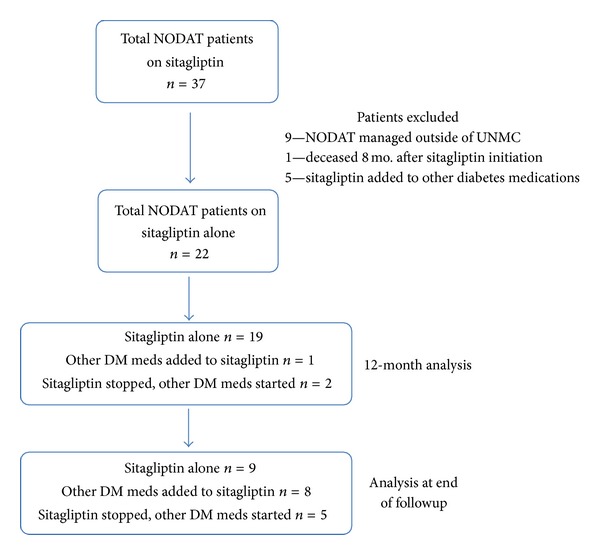
Flowchart outlining patient selection process. After patient exclusions, 22 patients were analyzed both at 12 months after initiation of sitagliptin and at the end of followup. DM = diabetes mellitus.

A total of 65 patients were identified to have received sitagliptin after kidney transplant during the study dates. Of these, 37 patients met criteria for NODAT (Figure 1). Of these 37 patients, a total of 15 were excluded from the study: 9 patients who obtained their diabetes care at outside clinics and clear records of diabetes medication adjustments were not available; 1 patient who was deceased 8 months after sitagliptin initiation; and 5 patients because sitagliptin was added to other diabetes medications. The remaining twenty-two (22) patients met inclusion criteria and had at least one year of followup and were subsequently analyzed. This cohort of 22 patients does include long-term followup of eight of the fifteen patients analyzed previously at our center in a 3-month prospective study of sitagliptin for the treatment of NODAT [18]. Baseline characteristics of the 22 patients included in the study are shown in Table 1. Of the 22 patients, NODAT was diagnosed in 21 by fasting blood glucose ≥7.0 mmol/L on two occasions; one patient was diagnosed with random blood glucose >11.1 mmol/L with symptoms of diabetes. A majority of patients utilized tacrolimus-based immunosuppression with the addition of either sirolimus or mycophenolate mofetil (Table 1). No patients received chronic corticosteroids during the follow-up period. Hepatitis C has been reported to be a risk factor for NODAT; however, none of our subjects were hepatitis C positive [1]. Sitagliptin was the initial diabetes medication for a majority of the patients (16/22).

### 3.1. Short-Term (12-Month) Followup

**Figure 2:**
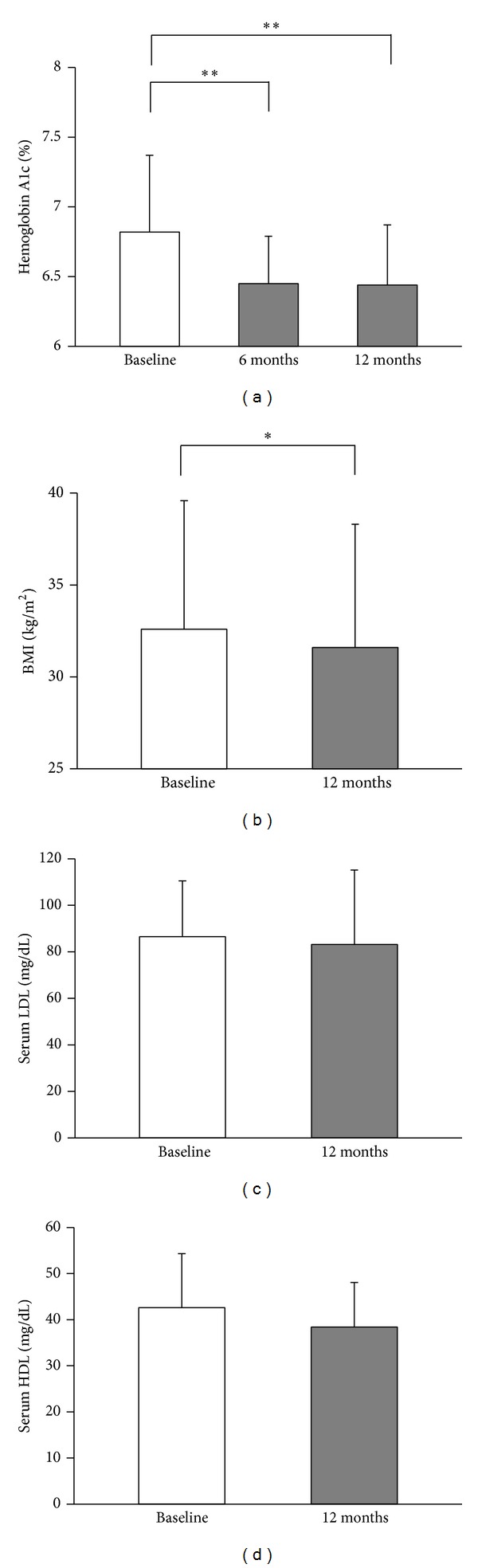
Sitagliptin improves NODAT control and is associated with a decline in BMI but no effect on serum lipids. (a) Diabetes control as measured by hemoglobin A1c for the entire cohort (*n* = 22) at 6 and 12 months after initiation of sitagliptin. (b) BMI for the entire cohort (*n* = 22) at start of sitagliptin compared to 12-month followup. (c), (d) Serum LDL-cholesterol and HDL-cholesterol at start of sitagliptin compared to 12-month followup. Data shown as mean + SD. **P < 0.01, *P < 0.05.

A total of twenty-two patients were analyzed at the end of 12 months of sitagliptin therapy. Of these, 19 patients remained on sitagliptin alone as their only diabetes medication, one patient required initiation of an additional oral diabetes medication, and 2 patients had sitagliptin discontinued in favor of other diabetes medications due to hyperglycemia. Diabetes control, as noted by HbA1c, was significantly improved at the end of 6 months and this effect persisted at 12 months (Figure 2(a)). Significance remained when analyzing just those patients (*n* = 19) who remained on sitagliptin alone for the entire 12 months (data not shown). There was a modest but significant decrease in BMI from the start of sitagliptin to the 12-month followup (Figure 2(b)), though certainly this weight loss may have been due to multiple factors including lifestyle modifications. LDL and HDL cholesterol values remained unchanged with sitagliptin therapy (Figures 2(c) and 2(d)).

**Figure 3:**
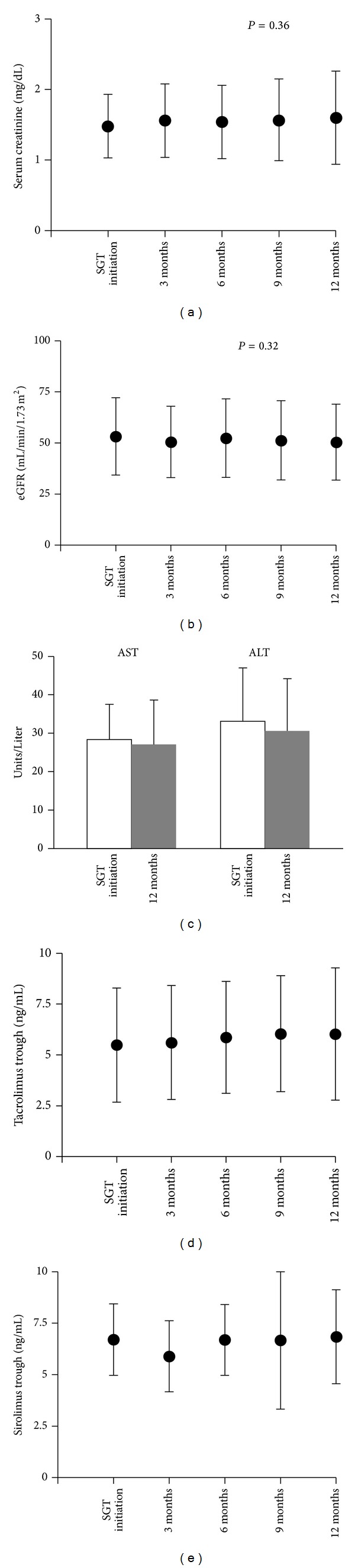
Sitagliptin use does not adversely affect renal graft function (as measured by serum creatinine and estimated GFR), liver function (as measured by serum AST and ALT), or immunosuppressant levels. (a) Serum creatinine. (b) Estimated GFR (eGFR). (c) Serum AST and ALT levels (*P* = NS). (d) Serum tacrolimus trough levels (*P* = NS). (e) Serum sirolimus trough levels (*P* = NS). Data shown as mean ± SD. SGT = sitagliptin.

At the 12-month followup, graft function as noted by serum creatinine and estimated GFR (eGFR) was no different than at start of sitagliptin (Figures 3(a) and 3(b)). We also observed no effect on liver transaminase levels (Figure 3(c)). We then reviewed immunosuppressant levels over the 12 months of followup and also meticulously reviewed medical records for immunosuppressant dose changes and, if changes to dose were made, the impetus for doing so. Tacrolimus and sirolimus levels remained stable throughout the 12-month followup (Figures 3(d) and 3(e)). In tacrolimus-treated patients, 1 patient had fluctuating levels and required a dose adjustment due to medication noncompliance. For the remainder of tacrolimus-treated patients only 3 patients required a single protocol dose adjustment and the rest maintained consistent tacrolimus doses. Similarly, the patient with noncompliance required frequent sirolimus dose adjustments during the follow-up period while 3 patients had single, protocol-related sirolimus dose adjustments.

### 3.2. Long-Term Followup

**Figure 4:**
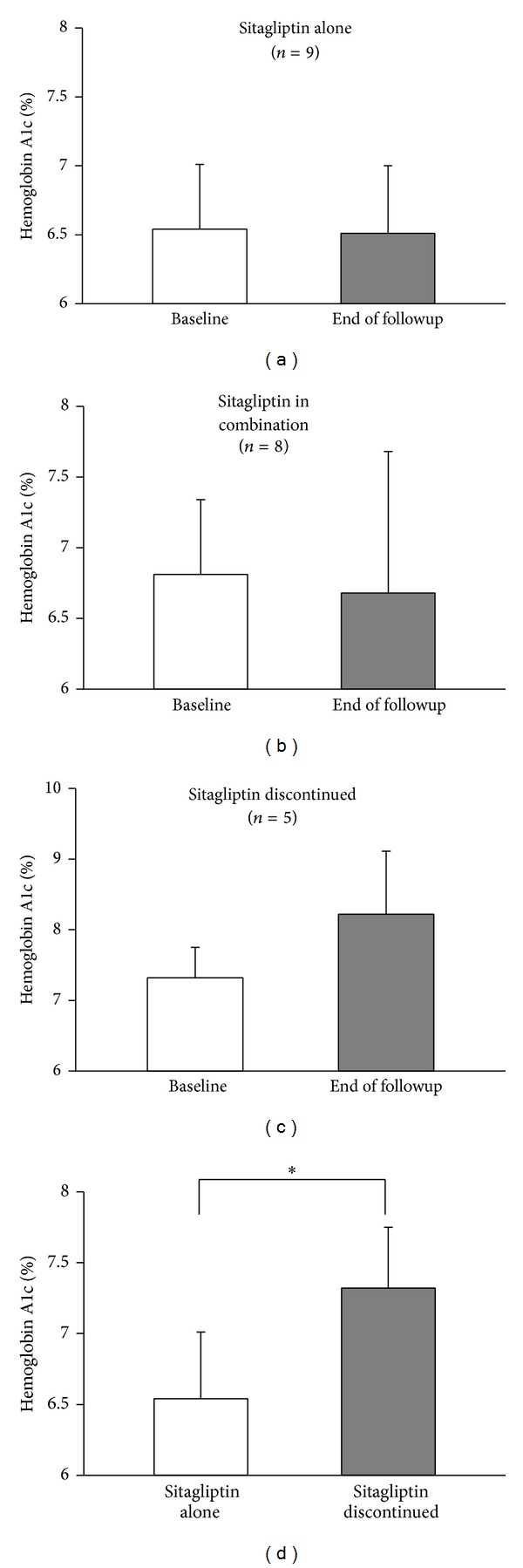
Sitagliptin, alone or in combination with other diabetes medications, can promote glycemic control in kidney transplant recipients with NODAT. Diabetes control at baseline compared to end of followup in (a) patients maintained on sitagliptin alone (*n* = 9, 31.8 ± 18.7 months of followup), (b) patients maintained on sitagliptin in combination with other diabetes medication(s) (*n* = 8, 44.9 ± 10.9 months of followup), and (c) patients who discontinued sitagliptin in favor of other diabetes medications (*n* = 5, 14 ± 4.2 months of followup). (d) Comparison of baseline hemoglobin A1c in sitagliptin alone patients (*n* = 9) compared to patients who discontinued sitagliptin in favor of other diabetes medications (*n* = 5). Data shown as mean ± SD.

To assess the long-term efficacy and risk for adverse effects of sitagliptin in kidney transplant recipients, we followed our cohort from the initiation of sitagliptin until December 2012 or discontinuation of sitagliptin, whichever came first (mean followup for all 22 patients = 32.5 ± 17.8 months). In the cohort, 17/22 patients remained on sitagliptin throughout the study period, for a mean duration of sitagliptin use of 37.9 ± 16.5 months. Of these 17 patients, 9 remained on sitagliptin alone for the entirety of the study (31.8 ± 18.7 months). Diabetes was well controlled in this group, with HbA1c maintained below 7% (6.5 ± 0.5%) at the end of followup (Figure 4(a)).

Eight patients continued on sitagliptin for the duration of the study (44.9 ± 10.9 months) but required addition of other diabetes medications to maintain glycemic control (oral agents, *n* = 4; insulin, *n* = 4). HbA1c remained well controlled in this group (Figure 4(b)) and two of the four patients on insulin required only basal insulin in addition to sitagliptin. Therefore, 6/8 patients requiring additional medications required only modest intensification of their diabetes medication regimen.

Five patients discontinued sitagliptin in favor of other diabetes medications after 14 ± 4.2 months. Of these, 4 patients discontinued sitagliptin due to worsening hyperglycemia (Figure 4(c)) and need for intensive insulin therapy while one patient was switched from sitagliptin to a sulfonylurea due to cost.

Hemoglobin A1c was significantly lower at baseline in patients who were able to continue on sitagliptin alone for the duration of the study (*n* = 9) compared to patients who discontinued sitagliptin and were switched to more aggressive diabetes management (*n* = 5) (Figure 4(d)). Change in BMI, from sitagliptin initiation to the end of followup, was no different between these groups (sitagliptin alone: −0.8 ± 2.2 kg/m^2^ versus sitagliptin discontinued: −1.1 ± 1.7 kg/m^2^, *P* = NS) suggesting the need for intensification of diabetes therapy in the sitagliptin discontinuation group was not necessarily related to weight gain.

Glucagon-like peptide-1 (GLP-1) therapies have been implicated to potentially increase the risk of pancreatitis, a matter of significant controversy. In the current study, no episodes of pancreatitis were reported during the follow-up period. Additionally, other transplant-specific adverse events were rare in our cohort, including one episode of acute rejection (47 months after sitagliptin initiation); two episodes of opportunistic infections (13 and 14 months after sitagliptin initiation); no graft loss; and one death (due to end-stage liver disease).

## 4. Discussion

Our study represents the largest cohort investigating the safety and efficacy of sitagliptin for the treatment of NODAT in kidney transplant recipients. Though our study was retrospective, through meticulous review of the medical records of patients followed up primarily in our transplant endocrine clinic we were able to closely follow medication prescribing changes, laboratory values, and reports of potential side effects and transplant-related comorbid events. In our cohort, sitagliptin was efficacious with a majority of patients meeting a goal HbA1c <7%. Specifically, sitagliptin alone was adequate to improve and maintain glycemic control in a majority of patients (19/22) for 12 months after sitagliptin initiation, consistent with its efficacy in the nontransplant population [21, 22].

Sitagliptin inhibits DPP-4 with several subsequent effects that improve blood glucose control including potentiation of insulin secretion and inhibition of glucagon secretion. In studies utilizing animal models of type 2 diabetes, sitagliptin has also been shown to reduce islet inflammation and protect beta cell mass [23–25]. Sitagliptin may also reduce amyloid-associated beta cell loss in type 2 diabetes [26]. Beta cells exposed to GLP-1 in vitro are resistant to toxicities associated with immunosuppressants, suggesting another potential benefit to DPP-4 inhibitor therapy for the treatment of NODAT [27]. These beta cell-specific protective effects of sitagliptin may explain why, in our cohort, patients with lower baseline HbA1c responded better to sitagliptin monotherapy as these patients may have been earlier into the disease and may have had more beta cell mass that was subsequently protected by sitagliptin. Overall, these findings suggest that initiating sitagliptin therapy early in the treatment of NODAT, when average glucose levels are only modestly elevated, may provide long-term benefits in terms of glycemic control. Larger cohorts are needed to investigate this point further.

In solid organ transplant recipients, drug-drug interactions and adverse effects of medications on graft function are of paramount importance when considering medications to treat comorbid conditions. We set out to better define the safety profile of sitagliptin in kidney transplant recipients in terms of effects on immunosuppressant levels/dosing, graft function, and other side effects. Renal function and tacrolimus and sirolimus dosing and trough levels remained stable during the initial 12-month follow-up period, indicating sitagliptin does not adversely affect renal function or interfere with immunosuppressant dosing. This is consistent with and extends upon similar data published from our center [18]. Hypoglycemia was not encountered in our cohort, consistent with reports of sitagliptin use in the nontransplant population.

The use of DPP-4 inhibitors for the treatment of hyperglycemia and NODAT in kidney transplant recipients is gaining more interest. Recently, a 3-month clinical trial of vildagliptin, another oral DPP-4 inhibitor, revealed that this agent improved oral glucose tolerance test outcomes and modestly improved hemoglobin A1c in kidney transplant recipients with impaired glucose tolerance (IGT) [14]. Similarly, a small, randomized control trial recently revealed vildagliptin to be a safe and effective therapy for the treatment of NODAT in kidney transplant recipients [28]. Finally, a 24-week retrospective study of linagliptin suggests this agent may be beneficial for treating NODAT, as well [29]. However, data regarding sitagliptin for the treatment of NODAT in kidney transplant recipients is scarce. A single pilot study of 15 patients followed up for 3 months revealed no significant effect of sitagliptin on renal function (as measured by estimated GFR) or tacrolimus or sirolimus levels [18]. Additionally, Iuppa et al. presented an abstract discussing the use of sitagliptin in solid organ transplant recipients and, although most of the subjects were liver transplant recipients, 18 kidney transplant recipients were reported on with a median followup of all patients (kidney and liver transplant recipients) of 178 days [30]. These small trials and a case report of a single kidney transplant recipient treated with sitagliptin for 2.5 years encompass the entire literature available on this subject [31]. Our study provides the most person-years of followup of any trial of sitagliptin for the treatment of NODAT in kidney transplant recipients. Additionally, though retrospective, our study provides the longest followup of any DPP-4 inhibitor used for the treatment of NODAT in kidney transplant recipients.

Limitations of our study include the retrospective nature of our study and the small cohort size. However, large, prospective studies regarding glucose-lowering therapy for NODAT do not exist currently (as reviewed) and our study represents the largest study to date examining the use of sitagliptin in the setting of NODAT after kidney transplantation [32]. Our cohort was comprised mostly of Caucasian males and steroids were not part of the maintenance immunosuppressant regimen. These factors may limit the ability to generalize our findings. Finally, our mean time to diagnosis of NODAT (56.3 ± 57.7 months after transplant) was longer than many studies of NODAT. This likely reflects that the patients in our cohort had slower, less aggressive development of NODAT and did not require insulin therapy. Kidney transplant recipients with viable grafts remain at higher risk of diabetes development, a significant portion of the risk being ascribed to immunosuppressant therapy. Therefore, NODAT can conceivably develop at any time after transplant. Safe, effective therapies are needed for patients who develop NODAT regardless of the timing of the diagnosis, and sitagliptin may be useful for this purpose given the favorable side effect profile and lack of interaction with immunosuppressant medications.

## 5. Conclusions

In conclusion, we have shown that, in a small cohort of kidney transplant recipients who developed NODAT, sitagliptin was efficacious as a single agent or in combination with other glucose-lowering medications. Sitagliptin was also well tolerated and renal function and immunosuppressant levels and dosing were stable during 12 months of therapy. Sitagliptin may be valuable as a first-line agent in kidney transplant recipients diagnosed with NODAT who are candidates for oral therapy.

## Figures and Tables

**Table 1:** Baseline demographics of the study participants.

Demographics (*n* = 22)	
Gender (% male)	77
Race (% Caucasian)	86
Average age at transplant (years)	44.7 ± 11.8*
BMI at transplant (kg/m^2^)^#^	29.4 ± 5.5*
BMI at NODAT diagnosis (kg/m^2^)	32.2 ± 6.8*
Average time to NODAT diagnosis (mo.)	56.3 ± 57.7*
Donor type (% of patients)	
Deceased	50
Living, genetically related	32
Living, genetically unrelated	18
Immunosuppressants (number of patients/(%))	
Tacrolimus	19 (86)
Sirolimus	9 (41)
Mycophenolate mofetil	8 (36)
Cyclosporine	2 (9)
Mycophenolic acid	1 (4.5)
Azathioprine	1 (4.5)

*Values are mean ± SD.

^#^Data available on 19 of 22 patients.
